# miR-99b-targeted mTOR induction contributes to irradiation resistance in pancreatic cancer

**DOI:** 10.1186/1476-4598-12-81

**Published:** 2013-07-25

**Authors:** Feng Wei, Yan Liu, Yanhai Guo, An Xiang, Guangyi Wang, Xiaochang Xue, Zifan Lu

**Affiliations:** 1 Department of Hepatobiliary & Pancreas Surgery, the First Hospital, Jilin University, Changchun, China; 2 Institute of Military Veterinary Medicine, Academy of Military Medical Sciences, Changchun, China; 3 State Key Laboratory of Cancer Biology, Department of Pharmacogenomics, School of Pharmacy, the Fourth Military Medical University, Xi’an, China; 4 State Key Laboratory of Cancer Biology, Department of Biopharmaceutics, School of Pharmacy, the Fourth Military Medical University, Xi’an, China

**Keywords:** Radiation resistance, mTOR, AZD8055, Pancreatic cancer

## Abstract

**Background:**

Radiation exerts direct antitumor effects and is widely used in clinics, but the efficacy is severely compromised by tumor resistance. Therefore uncovering the mechanism of radioresistance might promote the development of new strategies to overcome radioresistance by manipulating activity of the key molecules.

**Methods:**

Immunohistochemistry were used to find whether mTOR were over-activated in radioresistant patients’ biopsies. Then Western blot, real-time PCR and transfection were used to find whether radiotherapy regulates the expression and activity of mTOR by modulating its targeting microRNA in human pancreatic cancer cell lines PANC-1, Capan-2 and BxPC-3. Finally efficacy of radiation combined with mTOR dual inhibitor AZD8055 was assessed *in vitro* and *in vivo*.

**Results:**

Ionizing radiation promoted mTOR expression and activation in pancreatic cancer cells through reducing miR-99b expression, which negatively regulated mTOR. Novel mTOR inhibitor, AZD8055 (10 nM, 100 nM, 500 nM) synergistically promoted radiation (0–10 Gy) induced cell growth inhibition and apoptosis. In human pancreatic cancer xenografts, fractionated radiation combined with AZD8055 treatment further increased the anti-tumor effect, the tumor volume was shrinked to 278 mm^3^ after combination treatment for 3 weeks compared with single radiation (678 mm^3^) or AZD8055 (708 mm^3^) treatment (P < 0.01).

**Conclusions:**

Our data provide a rationale for overcoming radio-resistance by combined with mTOR inhibitor AZD8055 in pancreatic cancer therapy.

## Background

Pancreatic cancer is the fourth leading cause of cancer death, and is amongst the deadliest of human cancers. Only 10-15% patients undergo surgery due to late diagnosis, therefore radiotherapy becomes the major way in the treatment of pancreatic cancers in clinics, either alone or in combination with chemotherapy [1]. Local control of tumor growth is partly achieved by radiation-induced cell death as a result of damage to cell membranes and DNA [2,3]. However, the efficacy of radiotherapy remains limited due to intense tumor resistance. The molecular mechanisms underlying radiation resistance of pancreatic cancer are not fully understood [4].

The mammalian target of rapamycin (mTOR), a well-known serine/threonine kinase, is identified as a downstream target of PI3K/Akt survival pathway and functions as a central regulator of cell growth, proliferation and survival [5,6]. Accumulating evidence demonstrated that mTOR was dysregulated in various cancers, its over-expression and over-activation contribute to cancer progression and drug-resistance [7,8]. As a result, mTOR inhibitors represent a promising therapeutic approach for cancer and solid tumors [9,10].

The first generation mTOR inhibitors, like rapamycin and its analogs everolimus (RAD001), temsirolimus (CCI-779) and ridaforolimus (AP23573), have been developed as cancer therapeutic agents [10,11]. However, they are insufficient for achieving a broad and robust anticancer effect due to the feedback of AKT activation via up-regulating insulin-like growth factor-1 (IGF-1) [12]. AZD8055, a novel ATP-competitive inhibitor of mTOR kinases, besides preventing feedback to AKT, potently showed excellent selectivity (about 1,000 fold) against all class I PI3K isoforms and other members of the PI3K-like kinase family. AZD8055 is currently tested in phase I clinical trials as an anti-tumor drug [13,14]. Prior studies reported that combination of mTOR inhibitor RAD001 with radiotherapy can delay solid tumor growth *in vitro* and *in vivo* due to synergistic anti-angiogenic and anti-vascular effects [15], but the detail mechanisms remain poorly defined. Here, we wonder whether mTOR inhibitor AZD8055 can also amplify the radiotherapeutic effects in pancreatic cancers.

MicroRNAs (miRNAs) are a class of small non-coding RNAs which play important roles in gene regulation by targeting mRNA in a sequence-specific manner, and their dysregulations are a common feature in tumorigenesis and drug-resistance [16,17]. Numerous studies have shown that miR-99b, miR-100, miR-199a-3p, miR-451, miR-144 and miR-101 can directly or indirectly mediate mTOR expression [18-23], and reduction of these miRNAs was connected with the elevated levels of mTOR in prostate cancer and endometrial carcinoma [18,24]. However, it is still not clear whether these miRNAs can be regulated by radiation and be connected with aberrant mTOR activation in pancreatic cancer.

In this study, we identified that mTOR is positively regulated by radiation in both human pancreatic biopsy specimens and cell lines, and this mTOR upregulation is promoted by radiation induced miR-99b downregulation. We further provided evidence that dual mTOR inhibitor AZD8055 significantly reversed the aberrant mTOR activation, consequently sensitized pancreatic cancer cell lines and xenografts to radiotherapy. Thus, our data provide a rationale for overcoming radio-resistance by combined with mTOR inhibitor AZD8055 in pancreatic cancer therapy.

## Results

### mTOR was upregulated in pancreatic cancer patients subjected to radiotherapy

**Figure 1:**
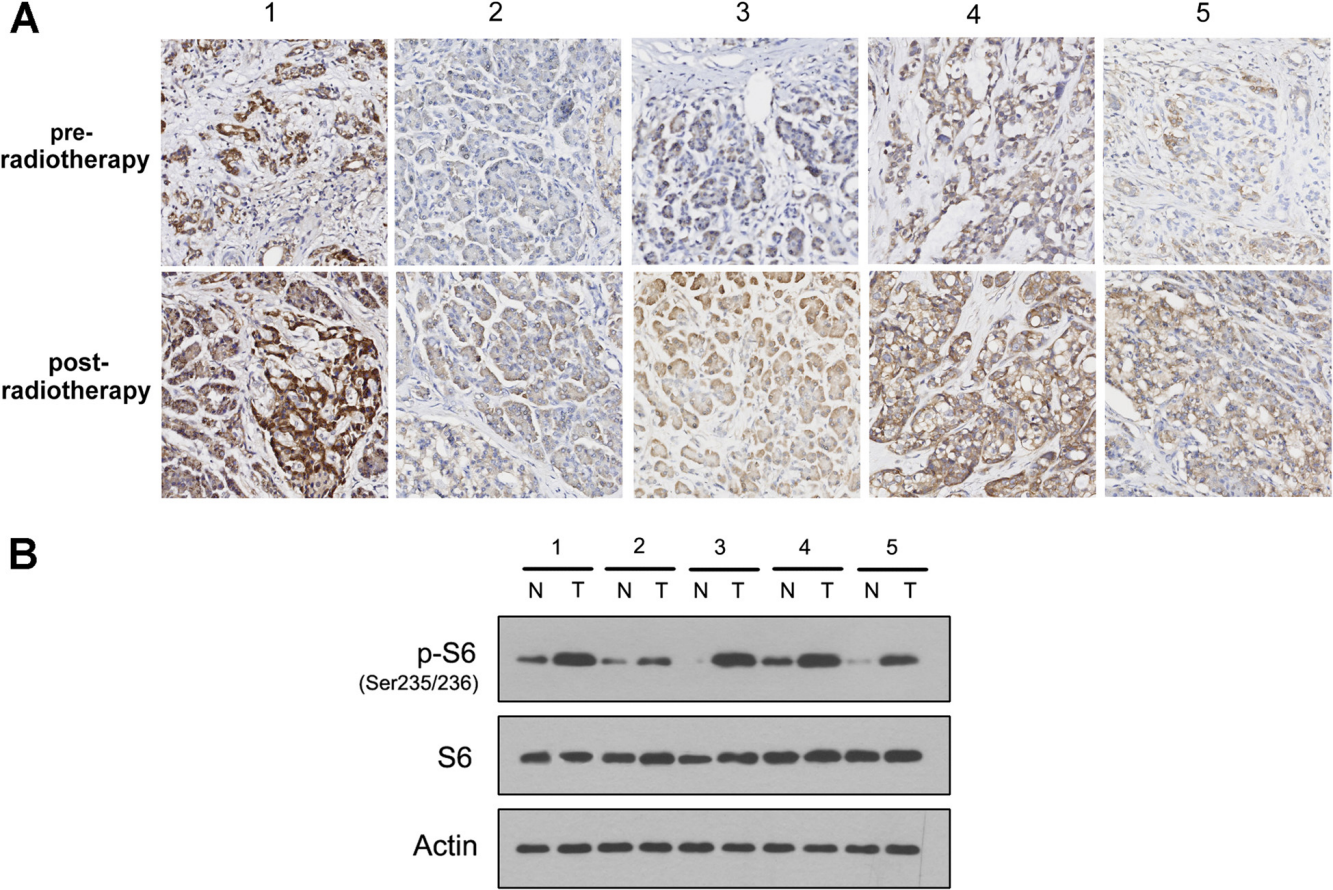
**mTOR expression and activation in pancreatic cancer patients before and after radiotherapy. (A)** Examination of mTOR in the tissue sections from pancreatic cancer patients pre- or post-radiotherapies by Immunohistochemistry (200 × magnification). **(B)** Western blot analysis of S6 and p-S6 protein in pre-radiotherapy tumors (N) and post-radiotherapy tumors (T). Actin was used as a loading control.

Although some signaling cascades such as Ras/PI3K/PTEN/Akt/mTOR, Ras/Raf/MEK/ERK and p53 have been implicated in regulation of tumor radioresistance, the detail mechanism is still largely unknown. To determine the key factors that influence the response of pancreatic cancer patients to radiotherapy, tumor biopsies from patients subjected to radiotherapy were examined. Several proteins, including mTOR, were differentially expressed in pre- or post-radiotherapy specimens. As shown in Figure 1, the expression of mTOR in post-radiotherapy samples was significantly higher than that in pre-treatment specimens by immunohistochemical analysis (Figure 1A). Western blot further confirmed that the level of active phosphorylated S6 (p-S6) as the key downstream molecule of mTOR signaling pathway was consistently up-regulated in the samples upon stimulation with radiation (Figure 1B). These data indicated that radiotherapy could induce the over-expression and over-activation of mTOR pathway in pancreatic cancer tissues and which may relate with the tumor resistance to radiotherapy.

### Ionizing radiation upregulates mTOR in pancreatic cancer cells at both transcriptional and protein levels

**Figure 2:**
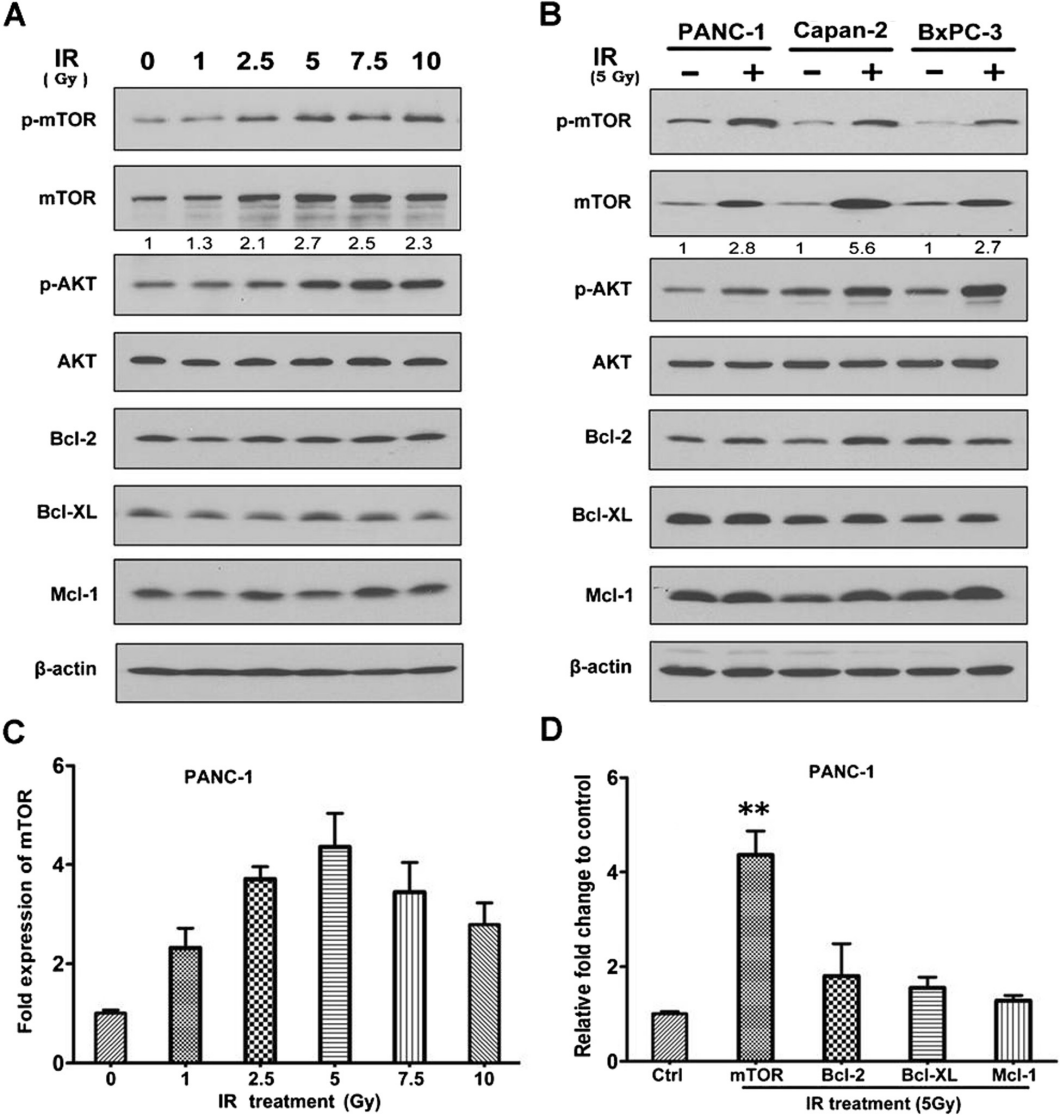
**Radiation induces mTOR phosphorylation in association with mTOR upregulation on mRNA and protein levels. (A)** PANC-1 cells were treated with indicated doses of IR for 1 h. mTOR, p-mTOR, AKT, p-AKT or Bcl-2 family members were analyzed by Western blot, and the intensity of mTOR expression was quantified with ImageJ software. **(B)** The above proteins were analyzed in PANC-1, Capan-2 and BxPC-3 cell lines after treated with radiation at 5 Gy. **(C)** Relatively fold changes of mTOR mRNA were analyzed by RT-PCR in PANC-1 cells. **(D)** Relatively fold changes of mTOR, Bcl-2, Bcl-XL and Mcl-1 mRNA were analyzed by RT-PCR. GAPDH were used as a blank control. Data are representative of three experiments. Error bars represent as mean± S.D. **, P < 0.01 vs. control.

To identify whether ionizing radiation modulates the expression and activity of mTOR in human pancreatic cancer, PANC-1 cells were cultured in normal condition and treated with increasing doses of radiation for 1 h. As shown in Figure 2A, radiation induced a dose-dependent increase of both mTOR and p-mTOR at doses from 0 Gy to 10 Gy. To confirm this, mTOR levels were also examined in other two pancreatic cell lines, Capan-2 and BxPC-3, with radiation treatment at 5 Gy and the similar results were obtained (Figure 2B). Furthermore, the mRNA level of mTOR was detected and results showed that mTOR transcript was up-regulated by radiation in PANC-1 cells and the peak value appeared at 5 Gy by 4.36 fold (Figure 2C), similar data were obtained in BxPC-3 and Capan-2 cells (data not shown). Meanwhile, Bcl-2, Bcl-XL and Mcl-1 as principal members of apoptosis family showed no big difference before and after radiation treatment (Figure 2D). Collectively, ionizing radiation significantly induces mTOR expression and activation at mRNA as well as protein levels, which possibly contribute to radioresistance in pancreatic cancer.

### mTOR is a critical factor in pancreatic cancer radioresistance

**Figure 3:**
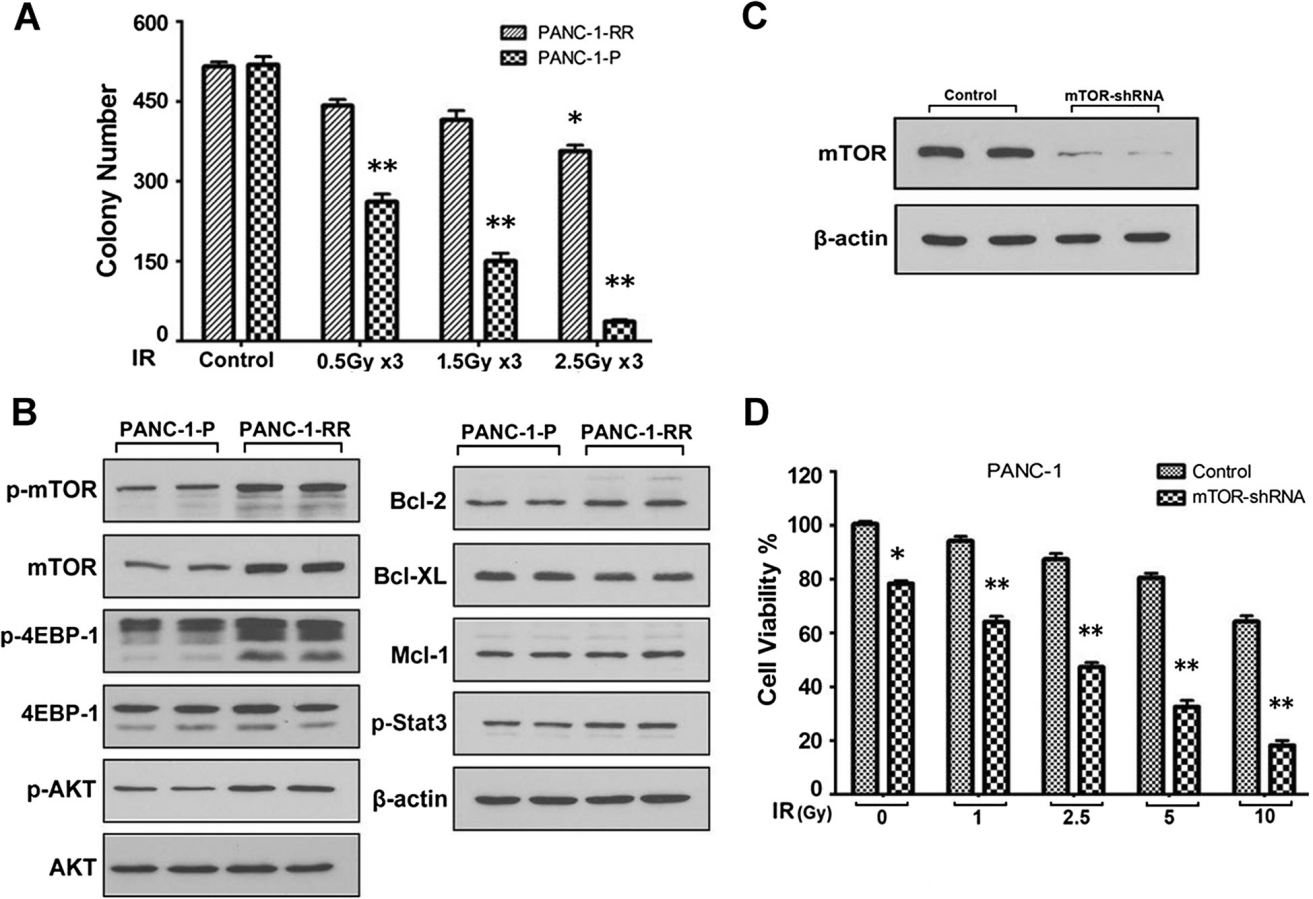
**mTOR play a critical role in pancreatic cancer radioresistance. (A)** Parental PANC-1 (PANC-1-P) and radiation resistant PANC-1 cells (PANC-1-RR) were treated with increasing doses of radiation and cultured for 10 days, cell growth was analyzed by colony formation assay and the data were statistically analyzed. The experiment was repeated three times and the number of colonies was presented as mean ± SD. *, P < 0.05 vs. control and **, P < 0.01 vs. control and PANC-1-RR cells. **(B)** Expression and/or phosphorylation of mTOR, Akt, 4EBP1, Bcl-2, Bcl-XL and Mcl-1 in PANC-1-P and PANC-1-RR cells were analyzed by Western blot. **(C)** mTOR shRNA and control were transfected into PANC-1 cells and mTOR expression was analyzed by Western blot. **(D)** Cells were transfected and then treated with increasing doses of radiation for 48 h, cell viability was analyzed by SRB assay. Error bars represent ± S.D. *, P < 0.05 vs. vector-only control and **, P < 0.01 vs. vector-only control.

To further verify whether mTOR is a direct factor that is involved in radioresistance of pancreatic cancer, PANC-1 irradiation-resistant cell line (PANC-1-RR) was generated and colony formation assay was used to confirm the radioresistance ability of PANC-1-RR (Figure 3A). Intriguingly, higher levels of mTOR and p-mTOR were observed in PANC-1-RR cells as compared with PANC-1-P cells (Figure 3B). To further test that mTOR is indispensable in the radioresistance,mTOR specific shRNA was transfected into PANC-1 cells. After transfection, cells were treated with radiation for 48 h, results revealed that endogenous mTOR in PANC-1 cells was remarkably downregulated (Figure 3C) and PANC-1 cells were more sensitive to radiation in mTOR shRNA transfection group as compared with the control shRNA group (Figure 3D). All these data collectively demonstrate that radiation induced mTOR expression and activation contributes to radioresistance and knockdown of endogenous mTOR effectively overcomes the radioresistance of pancreatic cancer cells.

### Downregulation of miR-99b, a key mediator of mTOR kinase, contributes to radiation induced mTOR upregulation

**Figure 4:**
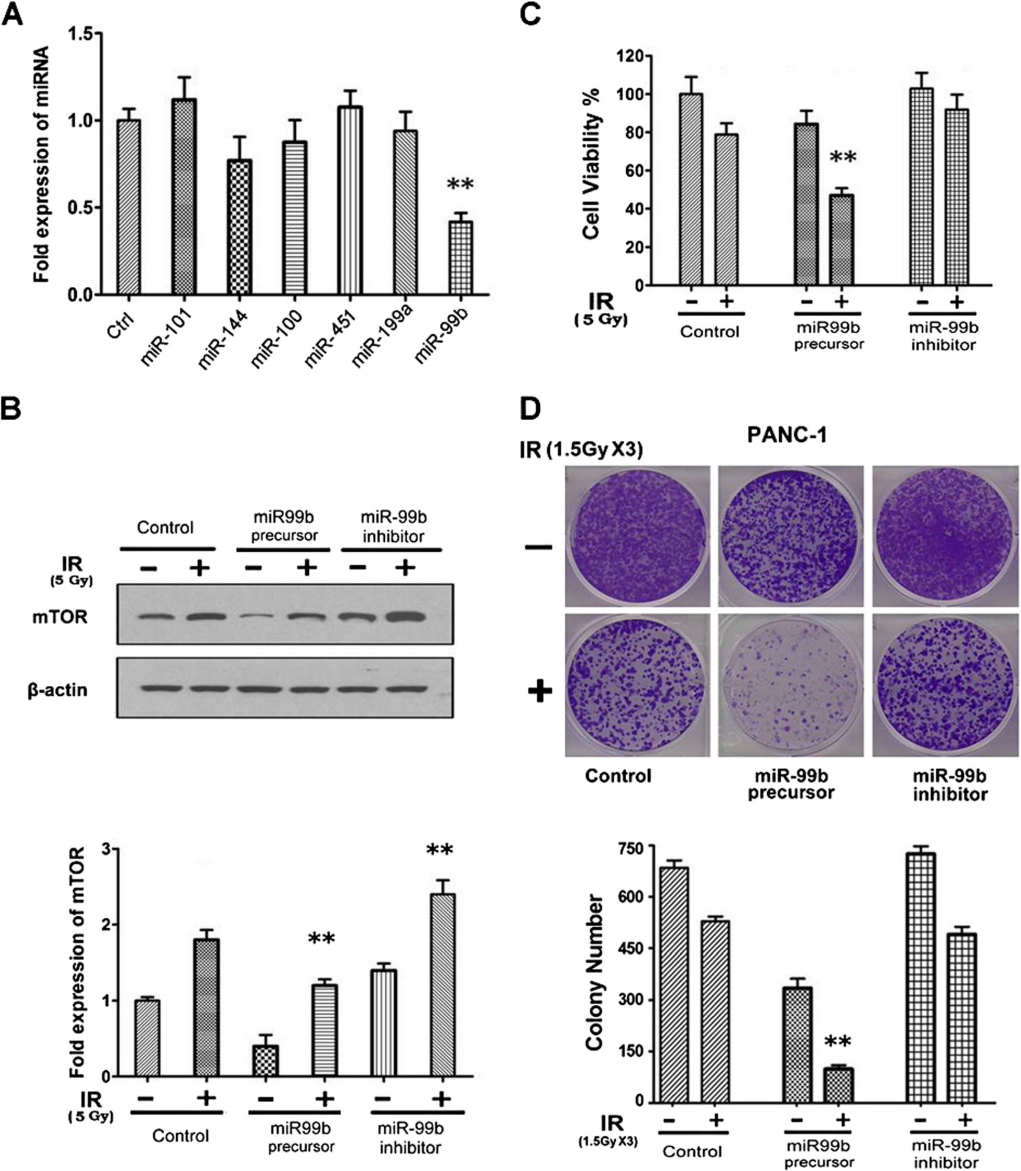
**Radiation down-regulates miR-99b which targets mTOR in pancreatic cancer cells. (A)** miR-101, miR-144, miR-100, miR-451, miR-199a and miR-99b were tested by real-time PCR before and after radiotherapy (5 Gy), **, P < 0.01 vs. control. **(B)** miR-99b precursor or miR-99b inhibitor was transfected into PANC-1 cells, followed by radiation at 5 Gy, and mTOR expression was analyzed by Western blot using an mTOR antibody. Western blot bands of mTOR were further quantified by the ImageJ software for calculating the expression of mTOR before and after miR-99b precursor/inhibitor transfection, **, P < 0.01 vs. control with IR. **(C)** PANC-1 cells were treated with radiotherapy (5 Gy) before and after miR99b precursor/inhibitor transfection for 48 h. Cell viability (%) was analyzed by SRB assay. Error bars represent as mean ± SD, **, P < 0.01 vs. control with IR or miR-99b inhibitor with IR. **(D)** PANC-1 cells were untreated or treated with radiation (1.5 Gy × 3) before and after miR-99b precursor/inhibitor transfection for 10 days and cell viability was analyzed by colony formation assay. The experiment was repeated three times and the number of colonies was represented as mean ± SD. **, P < 0.01 vs. control with IR or miR-99b inhibitor with IR.

It is well known that miRNAs widely participate in gene expression regulation and play critical roles in various physiological and pathological processes. To identify whether miRNAs were involved in radiation induced mTOR aberrant expression and activation, several miRNAs which targeted mTOR kinase including miR-101, miR-144, miR-100, miR-451, miR-199a and miR-99b were tested before and after radiation treatment. We found that miR-99b decreased most significantly by 2.7 fold after treatment with radiation at 5 Gy (Figure 4A). Although it was reported that mTOR was a target gene of miR-99b, we confirmed this with the luciferase reporter assay system and results showed that miR-99b can specifically recognize the seed sequence located in the 3′UTR of mTOR (Additional file 1: Figure S1). To further test whether miR-99b is able to regulate the expression of endogenous mTOR, miR-99b precursor or inhibitor was transfected into PANC-1 cells with or without radiation. Results showed that radiation dramatically upregulated mTOR expression in all these three groups compared with parallel samples without radiation, whereas miR-99b precursor suppressed and miR-99b inhibitor upregulated mTOR under the basal and radiation conditions when compared with control group (Figure 4B). All these findings disclose that reduction of miR-99b contributed to the upregulation of mTOR kinase in pancreatic cells and putatively influenced the cell sensitivity to radiotherapy.

In order to validate whether miR-99b could affect the cell sensitivity towards radiotherapy, PANC-1 cells were treated with radiation before and after miR99b precursor/inhibitor transfection. As shown in Figure 4C and D, cell growth and proliferation were significantly inhibited after downregulation of mTOR expression by miR-99b precursor whereas cells were more resistant to radiation after upregulation of mTOR by miR-99b inhibitor. All these data suggested that downregulation of miR-99b might induce cell resistance to ionizing radiation via enhanced mTOR expression.

### Inhibition of mTORC1/2 activity by AZD8055 sensitizes pancreatic cancer cells to ionizing radiation

**Figure 5:**
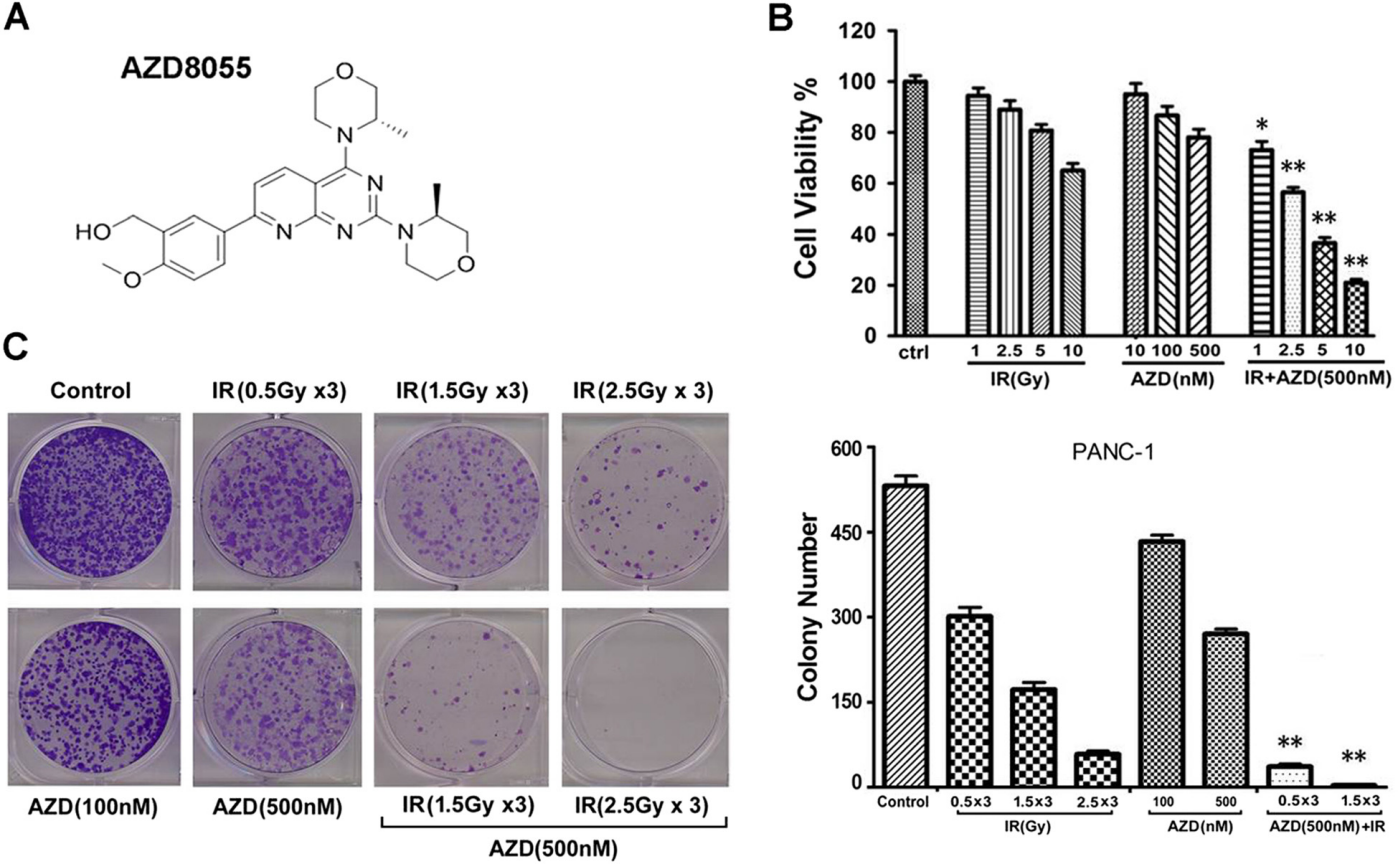
**AZD8055 blocks radiation-induced mTOR phosphorylation and sensitizes pancreatic cancer cells to radiotherapy. (A)** Chemical structure of mTOR dual inhibitor AZD8055, [5-[2,4-Bis((3S)- 3-methylmorpholin −4-yl)pyrido[2,3-d]pyrimidin-7-yl]-2-methoxyphenyl]methanol. **(B)** PANC-1 cells were treated with indicated doses of radiation in the absence or presence of AZD8055 for 48 h, Cell growth inhibition was analyzed by SRB assay. Error bars represent as mean ± SD. *, P < 0.05 vs. control or IR treatment alone and **, P < 0.01 vs. control or IR treatment alone. **(C)** PANC-1 cells were treated with radiation or AZD8055, alone or in combination, and cell viability was analyzed by colony formation assay. The experiment was repeated three times and the number of colonies was presented as mean ± SD. **, P < 0.01 vs. IR or AZD8055 treatment alone.

As we know, AZD8055 is a novel and effective ATP-competitive inhibitor of mTOR kinase activity (Figure 5A). It inhibits the phosphorylation of mTORC1 substrates S6K and 4E-BP1 as well as mTORC2 substrate AKT and downstream proteins. According to our above findings, we supposed that inhibition of mTORC1/2 phosphorylation by AZD8055 may enhance the anti-proliferative effect of radiation. To confirm this hypothesis, PANC-1 cells were treated with radiation in the absence or presence of AZD8055, the results disclosed that all of the doses of AZD8055 combined with radiation showed a synergetic inhibition of cell growth. As shown in Figure 5B, radiation (0 Gy, 1 Gy, 2.5 Gy, 5 Gy, 10 Gy) or AZD8055 (10 nM, 100 nM, 500 nM) single treatment caused less than 40% cell growth inhibition, whereas the combination caused more than 80%. Colony formation assay also showed that almost all the PANC-1 cells were eliminated by the combination treatment compared to radiation or AZD8055 treated alone (Figure 5C). The similar data were achieved with the other two pancreatic cancer cell lines (data not shown). Altogether, our data suggest that blockade of mTOR signal pathway by AZD8055 could reverse radioresistance and sensitize pancreatic cancer cells to ionizing radiation.

### AZD8055 enhances radiation induced cell cycle disruption and cell apoptosis

**Figure 6:**
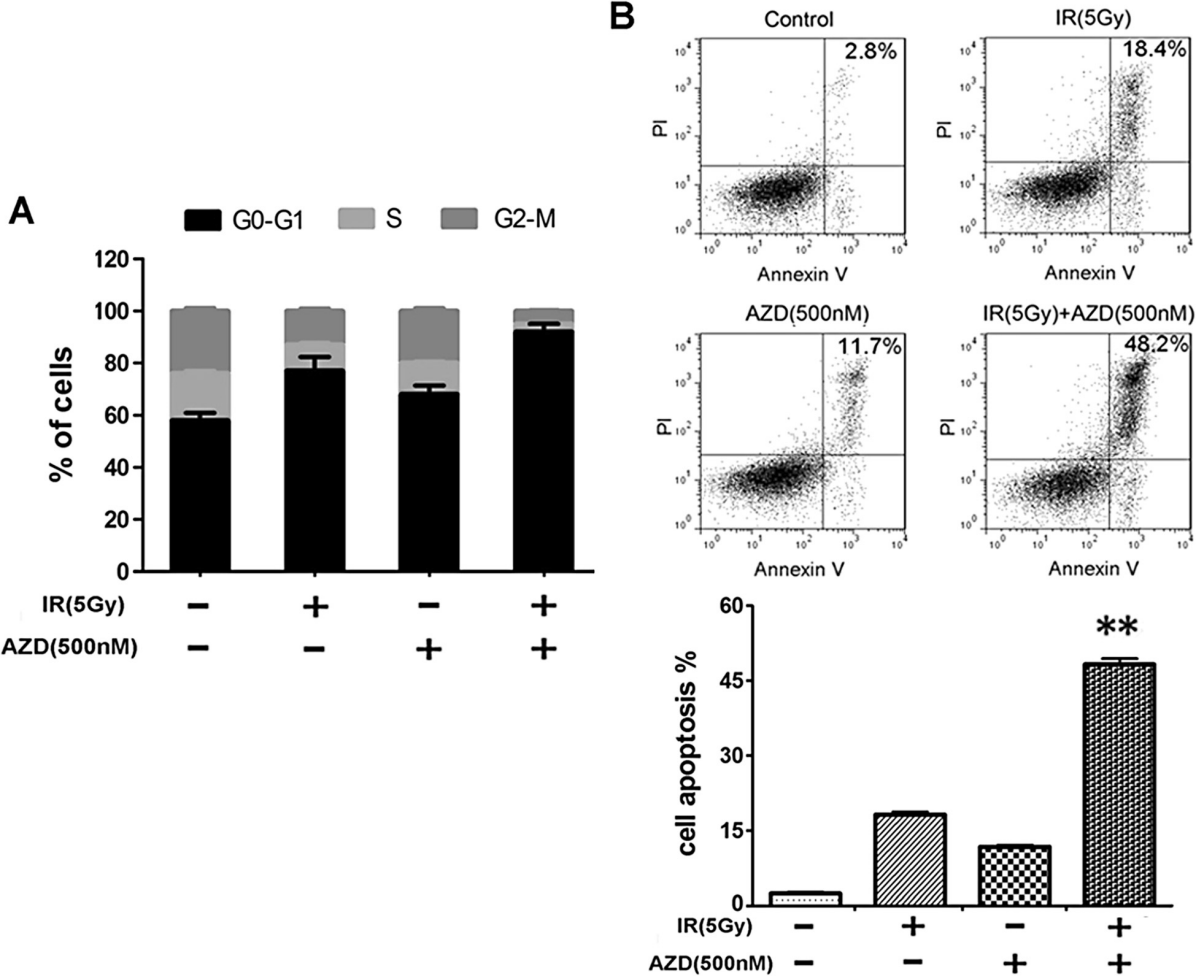
**Blockade of radiation-induced mTOR phosphorylation by AZD8055 enhances radiation induced cell cycle arrest and apoptosis. (A)** PANC-1 cells were treated with radiation (5 Gy) in the absence or presence of AZD8055 (500 nM) for 24 h, followed by analysis of cell cycle by flow cytometry. The cells in G0/G1, S and G2/M phases were presented in the percentage. The experiment was repeated three times and the percentages of cells in G0/G1 and S phases were presented as mean ± SD. **(B)** Annexin-V/PI cell apoptosis analysis of PANC-1 cells treated with radiation in the absence or presence of AZD8055 for 24 h, flow cytometry data showed the percentage of apoptotic cells The experiment was repeated three times and the percentage of apoptotic cells was presented as mean ± SD. **, P < 0.01 vs. IR treatment alone.

To evaluate whether AZD8055 combined with radiation affects cell cycle distribution, PANC-1 cells were treated with indicated doses of radiation and/or AZD8055 as described previously. We found that AZD8055 or radiation alone caused a slight accumulation of cells in G0/G1 phases (from 58% to 67% or 77%) and a mild reduction in S phase (from 18% to 10% or 8%) compared with control cells, whereas a more extensive cell cycle perturbation was caused by their combined treatment, with an accumulation of cells in G0-G1 phase (93%), and a significant reduction in S phase (3%) (Figure 6A).

Then Annexin V assay was employed to test whether the combination treatment was accompanied with increased programmed cell death. As shown in Figure 6B, Radiation or AZD8055 alone merely induced a small number of cells apoptosis by 18.4% or 11.7% even at 5 Gy or 500 nM. Intriguingly, AZD8055 combined with radiation synergistically induced significant cell apoptosis by 48.2%. Our findings indicate that AZD8055 enhanced ionizing radiation induced cell apoptotic and cell cycle arrest.

### Suppression of mTOR activation by AZD8055 enhances antitumor efficacy of radiation in pancreatic cancer xenografts

**Figure 7:**
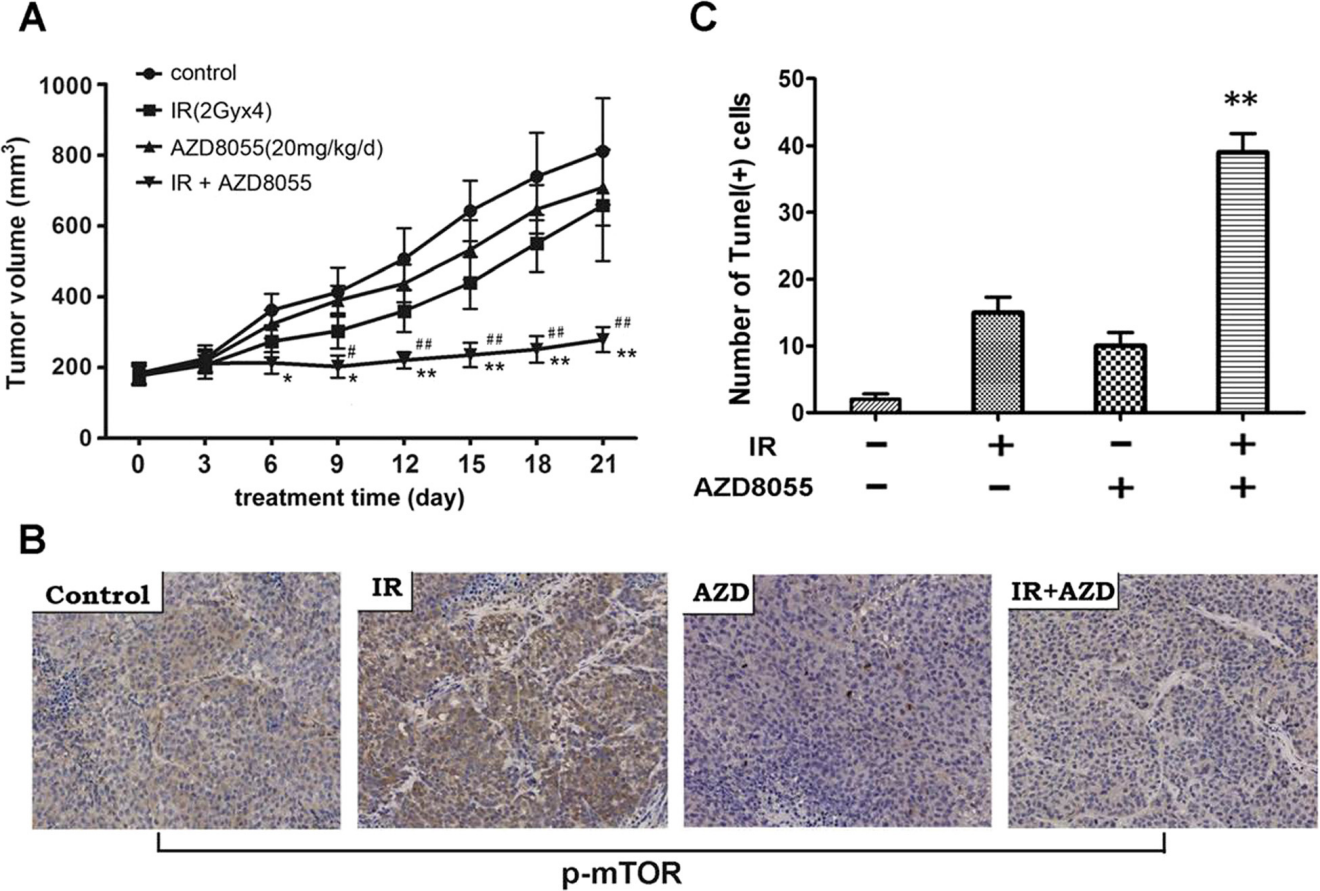
**Inhibition of radiation induced mTOR phosphorylation enhances antitumor efficacy of radiotherapy *in vivo*.** Four groups of Nu/Nu nude mice (n=10) with PANC-1 xenografts were treated as indicated. After 21 days treatment, the mice were sacrificed and the tumors were removed. **(A)** The tumor volumes from the same group mice were presented as mean ± SD and statistically analyzed. *, P < 0.05 and **, P < 0.01 vs. control and #, P < 0.05 and ##, p < 0.01 vs. radiation treatment alone. **(B)** The xenografts were embedded in paraffin blocks and paraffin sections were examined by immunohistochemistry using p-mTOR antibody. **(C)** Apoptosis in tumor tissues was analyzed by TUNEL assay using Tumor TACS^TM^*In Situ* Apoptosis Detection Kit. TUNEL positive cells were counted for every 1000 cells from ten areas of each slide, and three different slides were analyzed for each sample. Data represents as mean ± SD, **, P < 0.01 vs. IR treatment alone.

Our *in vitro* studies have proved the principle that radiation combined with AZD8055 could synergistically inhibit cell proliferation and induce apoptosis. To evaluate these effects *in vivo*, mice bearing subcutaneous PANC-1 xenografts were randomized and treated for three weeks as described in “Materials and methods”. As indicated in Figure 7A and B, in mice that received fractionated radiation alone, tumors grew slowly during the early two weeks, then the growth rate resumed similar to the control group (P > 0.05), meanwhile in association with high level of p-mTOR in tumor tissues. Interestingly, more cooperative antitumor effect was observed when AZD8055 was used in combination with fractionated radiation, with a significant reduction of the volumes of the xenografts at the end of treatment in all of the mice as compared with control and radiation alone group. In addition, AZD8055 apparently blocked radiation-stimulated mTOR expression and phosphorylation in tumor tissues (Figure 7B). All the data collectively demonstrated that blockage of radiation-induced aberrant mTOR expression and phosphorylation significantly sensitized pancreatic cancer cells to radiation and acquired increased anti-tumor activity *in vivo*.

To evaluate the role of apoptosis in this xenografts model, TUNEL assay was used to detect the tumor tissues and results showed that inhibition of mTOR pathway by AZD8055 significantly enhances apoptosis in pancreatic xenograft tissues (P < 0.01) (Figure 7C).

## Discussion

Pancreatic cancer is the most devastating type of cancer, the 5-year survival rate of patients is less than 5% [25]. Until now, the late diagnosis and persistent resistance to chemo- and radio-therapy are still the leading problems in clinics [26]. Although the current standard gemcitabine therapy and radiotherapy prolong the survival of patients with advanced pancreatic cancer for a few months, the high rate of recurrence still confused the clinical therapy [27,28].

As we know, radiation has been widely used for pancreatic cancer therapy because it can induce cell death by damaging cell membranes and DNA [29]. However, radiation is also able to stimulate some other important signaling pathways which regulate cell survival, proliferation and apoptosis [30,31]. Until now, it is unclear about which signaling pathway plays the key role in the radiotherapy for unresectable pancreatic cancer. By exploiting with the patient biopsy samples, we demonstrated that mTOR expression was significantly up-regulated in clinical radiotherapy tissues, suggesting that it may contribute to the clinical radiotherapy resistance. This data provided the direct *in vivo* clinical evidence supporting that radiation induced mTOR upregulation might in association with pancreatic cancer cell resistance to radiation. From the cell line data, we also observed mTOR over-expression and over-activation after radiotherapy. Considering that miRNAs participated in various physiological and pathological processes by directly regulating target genes expression, we purposely detected various putative miRNAs that may repress mTOR and miR-99b was found to be down-regulated by radiation. Not surprisingly, mTOR was reversely regulated when miR-99b was overexpressed or knocked down under both basal and radiation conditions. In addition, cell sensitivity to radiotherapy was also influenced by miR-99b. Our results not only provide some new clues for mTOR upregulation in radiation-treated pancreatic clinical samples and cell lines, but also demonstrated that miR-99b played important roles in pancreatic cancer radioresistance and maybe a candidate therapeutic target for pancreatic cancer.

Considering mTOR was up-regulated by radiation through miR-99b and mTOR signal pathway plays critical roles in regulating cancer cell survival, proliferation and apoptosis, we wonder whether mTOR inhibition have synergistic effects with radiotherapy. AZD8055, an mTORC1/C2 dual inhibitor, was employed to inhibit mTOR activity and block the feedback activation of AKT. Results demonstrated that AZD8055 treatment significantly potentiates the cytotoxic effects of ionizing radiation in human pancreatic cancer cell lines. Additionally, we also confirmed that the growth inhibition was accompanied by a perturbation of cell cycle with the marked reduction of cells in S phase and an accumulation in G0/G1 phase. Moreover, AZD8055 treatment enhanced radiation induced cell apoptosis. Intriguingly, these events were paralleled by suppressing the expression and function of mTOR, but do not influence the anti-apoptotic family members such as Bcl-2, Bcl-XL and Mcl-1, suggesting that AZD8055 and radiation synergistically induced cell apoptosis through mTOR related signaling pathways but not Bcl-2 family in pancreatic cancer cells.

Similar to *in vitro* results, the growth of pancreatic cancer xenografts was also inhibited by fractionated radiotherapy or application of AZD8055 *in vivo,* and surely combination of AZD8055 and radiotherapy suppressed growth of PANC-1 xenografts more effectively than treatment with either therapy alone. On the whole, inhibition of mTOR activity by AZD8055 effectively reversed radio-resistance both *in vitro* and *in vivo*. Therefore inhibiting mTOR activity by AZD8055 may be an effective way to overcome radioresistance and potently sensitize pancreatic cancers to radiation.

In summary, our study observed mTOR upregulation in clinically treated biopsy samples and identify a novel mechanism related with mTOR upregulation in pancreatic cancer cells after radiation therapy. miR-99b reduction was involved in mTOR upregulation and therefore affected the radiotherapy sensitivity of pancreatic cancer cells. Blockade of mTOR by AZD8055 represents a new therapeutic strategy to overcome radioresistance in patients with pancreatic cancer.

## Conclusions

In conclusion, the results of this study demonstrate the upregulation of mTOR by radiation via downregulating miR-99b and provide the first evidence of the regulatory effects of radiation on mTOR expression and activation. We propose that mTOR play a critical role in radioresistance and its dual inhibitor AZD8055 can be used in combination with radiation to overcome the radioresistance in pancreatic cancer treatment.

## Materials and methods

### Materials

AZD8055 was purchased from Selleck Chemicals (Houston, TX, USA). Antibodies for mTOR, p-mTOR, Akt, p-Akt (S473), S6 and p-S6 (Ser235/236) were purchased from Cell Signaling Technology (Beverly, MA). Bcl-2, Bcl-XL and Mcl-1 antibodies were from Santa Cruz Biotechnology (Santa Cruz, CA). Tumor TACS™ *In Situ* Apoptosis Detection Kit was purchased from Trevigen, Inc. (Gaithersburg, MD). mTOR shRNA was obtained from Sigma-Aldrich (St. Louis, MO). All other reagents were obtained from stated commercial sources.

### Biopsies collection of pancreatic cancer patients

Patients with locally advanced pancreatic cancer were diagnosed by computed tomography (CT) and MRI imaging, and all patients received a comprehensive evaluation and were considered to be unresectable. Eight patients were treated with Intensity-modulated radiation therapy (IMRT) at 50 Gy and responses were evaluated via computed tomography. Five patients who have stable disease (SD) or progressive disease (PD) were resistant to IMRT among total 8 patients. The biopsies were taken by tru-cut needle from these five radiotherapy resistant patients. None of the subjects received other biotherapy or chemotherapy treatments. The study was approved by the ethics committees of the First Hospital of Jilin University and the Fourth Military Medical University. Written informed consents were also obtained from all subjects before study.

### Cell culture and sulforhodamine B assay

Human pancreatic cancer cells PANC-1, Capan-2 and BxPC-3 purchased from National Rodent Laboratory Animal Resource (Shanghai, China) were grown as previously described [32]. Briefly, these cell lines were cultured and maintained in exponential growth in Dulbecco’s modified Eagle’s medium (DMEM) containing 100 IU/ml penicillin, 100 μg/ml streptomycin, 20 mM glutamine and 10% heat-inactivated FCS (Atlanta Biologicals, Lawrenceville, GA) in a humidified atmosphere of 5% CO_2_ at 37°C. For sulforhodamine B (SRB) assay, the exponential growing cells were seeded at 6–8 × 10^3^/well in 96-well plates and cultured overnight. Cells were treated with radiation alone or combined with AZD8055. AZD8055 was added to cultured cells and radiation was applied 4 h later in single doses of 1, 2.5, 5 or 10 Gy. The cells were irradiated using an X-ray machine (X-RAD 320, Precision X-ray) at 320 kV, 10 mA with a 2-mm aluminum filter, and the dose rate was 2 Gy/min. Cells were then cultured at 37°C for 48 h and the surviving fractions were determined using SRB assay as previously described [33,34]. The absorbance was measured with a spectrophotometer (Bio-Rad Inc) at 510 nm and cell growth inhibition was calculated by using the equation: cell viability (*%*) = (At/Ac) × 100*%*, in which At and Ac represent the absorbance in treated and control cultures respectively, as described previously [12].

### Cell lysate and Western blot assay

Cells were lysed in ice-cold EBC buffer (50 mM pH 8.0 Tris, 120 mM NaCl, 0.5% NP-40, 50 mM NaF, 1 mM phenylmethylsulfonyl fluoride (PMSF), 20 μM sodium orthovanadate, 1 × Protease Inhibitors, 1 × Phosphatase Inhibitors) and proteins were quantified and subjected to SDS-PAGE electrophoresis, followed by protein transfer to nitrocellulose membranes. The membranes were incubated with the primary and secondary antibodies, then developed by chemiluminescence [35].

### RNA isolation and quantitative real-time PCR

Total RNA was isolated from cells using Trizol (Invitrogen), 1–10 μg of RNA was used to synthesize cDNA with SuperScript II First-Strand Synthesis System (Invitrogen) or TaqMan® MicroRNA Reverse Transcription Kit (Applied Biosystems). Aliquots of the reaction mixture were used for real-time PCR with Power SYBR Green PCR Master Mix or with the TaqMan® 2 × Universal PCR Master Mix. The reaction conditions: 50°C for 20 s, 95°C for 10 min followed by 40 cycles of 95°C for 15 s, 60°C for 1 min. All real-time PCR experiments were performed in triplicate. A melting curve was obtained to verify the presence of a single amplicon. The primer sequences are as described previously [36-38].

### Colony formation assay

PANC-1 cells were seeded in 6-well-plates (1000 /well), and then treated or untreated with radiation and AZD8055, alone or in combination. The medium was replaced with fresh medium containing the reagent and radiation-treatment every three days. After 10 days treatment, the medium was removed and cell colonies were stained with crystal violet (0.1% in 20% methanol). Pictures were taken using a digital camera to record the result as described [9]. To evaluate the colony formation ability of irradiation-resistant cells, PANC-1 irradiation-resistant cell line (PANC-1-RR) was firstly generated by plating PANC-1 cells in 100-mm culture dishes and irradiating with 2 Gy X-ray every three days over a period of 5 months, for a total dose of 100 Gy, and then colony formation assay was used as above mentioned [39].

### Transfection

PANC-1 cells were suspended in DMEM supplemented with 10% FBS and seed in 6-well plates (1 × 10^6^/well) and transfected with miR-99b precursor or inhibitor (Ambion) with Lipofectamine™ 2000 (Invitrogen) according to the manufacturer’s instruction. After 48 h of transfection, cells were treated by radiation at 5 Gy, then harvested and lysed for Western blot assay [40]. For mTOR interfering, mTOR shRNA with the sequence of CCGGGCTGTGCTAC ACTACAAACATCTCGAGATGTTTGTAGTGTAGCACAGCTTTTTG was used to transfect PANC-1 cells.

### Apoptosis analysis

Annexin V/PI Apoptosis Detection kit (Clontech Laboratories) was used for quantification of apoptosis. Cells were seeded in 6-well plates in the absence or presence of AZD8055 (500 nM), then radiation was applied 4 h later. After cultured for 24 h, 0.5-1 × 10^6^ cells were collected into each tube and gently washed with PBS. Cell pellets were suspended in 1 × binding buffer and stained with Annexin V and PI. After incubated for 15 min at RT in the dark, the apoptosis analysis was carried out using a FACScan (BD Biosciences) and analyzed using FlowJo software (Tree Star Inc).

### Cell cycle analysis

Cells were synchronized by growing in serum free medium for 48 h and then released into the cell cycle by adding 10% FBS to the medium. The cells were treated with radiation in the absence or presence of AZD8055 (500 nM) for 24 h, harvested, fixed with 70% ethanol, and stained with PI. Data were acquired using flow cytometry and analyzed using FlowJo software.

### Pancreatic cancer xenografts and treatments

Animal experiments were careful to follow the protocols approved by Jilin University and the Fourth Military Medical University Institutional Animal Care and Use Committees. PANC-1 cells (7 × 10^6^) were resuspended in HBSS and injected subcutaneously into the flank region of 6-week-old female athymic (nu/nu) mice (Shanghai, China). The tumors were allowed to grow to average volume of 200 mm^3^ prior to initiation of therapy as described [41]. Then mice were assigned randomly to four groups (n =10) as following: (1) vehicle control (5% DMSO, 100 μl/d p.o.); (2) 8 Gy fractionated radiotherapy (2 Gy for every three days); the radiation was performed using the same X-ray machine with a different filter (1.5 mm aluminum, 0.8 mm tin, and 0.25 mm copper), at a dose rate of 1 Gy/min; (3) AZD8055 (20 mg/kg/d), AZD8055 was dissolved in DMSO and administered by oral gavage (0.1 ml/10 g of body weight); (4) Combination of AZD8055 (20 mg/kg/d) and 8 Gy (2Gy × 4) fractionated radiotherapy. Tumor volumes were measured with a caliper every other day and calculated based on the formula: V = 4/3 × *π*(length/2 × (width/2)^2^). After 21 days treatment, mice were sacrificed and the tumors were removed and submerged in 10% neutrally buffered formalin for immunohistochemistry analysis.

### Immunohistochemistry

Four-μm thick paraffin sections were deparaffinised, rehydrated and stained using the R.T.U.Vectastain kit following the manufacturer’s standard protocol (Vector Laboratories). The sections were incubated with anti-mTOR antibody (1:50) overnight at 4°C, then stained with secondary antibody. Thereafter, the slides were exposed to DAB chromogen for 5 min, then hematoxylin counter stained, dehydrated, and treated with xylene following the approach as earlier reported [42]. Finally all slides were examined and representative pictures were taken using an Olympus BX41 microscope.

### TUNEL assay

TUNEL staining was performed by using Tumor TACS™ *In Situ* Apoptosis Detection Kit (Trevigen), the specimens were deparaffinised and labeled following the procedure provided by the manufacturer. Finally, DAB staining were visualized under microscopy [43]. For TUNEL assay, ten fields were randomly selected from each slide for measurement, the images were analyzed by MetaMorph software and presented as a percentage of the total number of cells [44].

### Statistical analysis

Levels of significance were determined by different methods, two-sided unpaired student’s t-test and one-factor ANOVA were used in the comparison between groups [45], and LSD-t tests was used in multiple comparisons. Results were considered statistically significant at P values < 0.05.

## Abbreviations

mTOR: Mammalian target of rapamycin; microRNA: miRNA; Gy: Gray; SRB: Sulforhodamine B

## Competing interests

The authors declare that they have no competing interests.

## Authors’ contributions

FW, YL, AX, YG and GW performed experiments; XX and ZL designed research, analyzed data, and edited the manuscript for intellectual content. All authors have made critical edits to the manuscript and have given final approval.

## Authors’ information

Feng Wei and Yan Liu are joint first authors.

## Supplementary Material

Additional file 1: Figure S1. mTOR is a target gene of miR-99b. **(A)** Sequence alignment of miR-99b with reverse complementary miR-99b (rcmiR-99b, as the positive control), mTOR, mutant rcmiR-99b (mrcmiR-99b) and mutant mTOR (mmTOR); mutant nucleotides are underlined. **(B-C)** Dual-luciferase reporter vectors were constructed with rcmiR-99b/mrcmiR-99b and mTOR-3′-UTR/mmTOR-3′-UTR cloned between *Not* I and *Xho* I sites in psiCHECK plasmid and murine macrophage RAW264.7 cells were transfected with the vectors alone or in the presence of miR-99b precursor or inhibitor. Vectors containing rcmiR-99b and mrcmiR-99b were used as controls. *Renilla* luciferase (RLuc) activity was measured and normalized to *Firefly* luciferase (FLuc), and recombinant vectors were normalized to empty vector. *P < 0.05 and **P < 0.01 vs. plasmid alone group. Data are representative of three experiments. Click here for file
