# Supplementary material for: miR-99b-targeted mTOR induction contributes to irradiation resistance in pancreatic cancer
Source: Mol Cancer. 2013 Jul 25;12:81. doi: 10.1186/1476-4598-12-81 (PMC3726417; doi:10.1186/1476-4598-12-81)
Supplement: Additional file 1: Figure S1. — mTOR is a target gene of miR-99b. (A) Sequence alignment of miR-99b with reverse complementary miR-99b (rcmiR-99b, as the positive control), mTOR, mutant rcmiR-99b (mrcmiR-99b) and mutant mTOR (mmTOR); mutant nucleotides are underlined. (B-C) Dual-luciferase reporter vectors were constructed with rcmiR-99b/mrcmiR-99b and mTOR-3′-UTR/mmTOR-3′-UTR cloned between Not I and Xho I sites in psiCHECK plasmid and murine macrophage RAW264.7 cells were transfected with the vectors alone or in the presence of miR-99b precursor or inhibitor. Vectors containing rcmiR-99b and mrcmiR-99b were used as controls. Renilla luciferase (RLuc) activity was measured and normalized to Firefly luciferase (FLuc), and recombinant vectors were normalized to empty vector. *P < 0.05 and **P < 0.01 vs. plasmid alone group. Data are representative of three experiments. [file 1476-4598-12-81-S1.doc]

**Supplementary information**

**
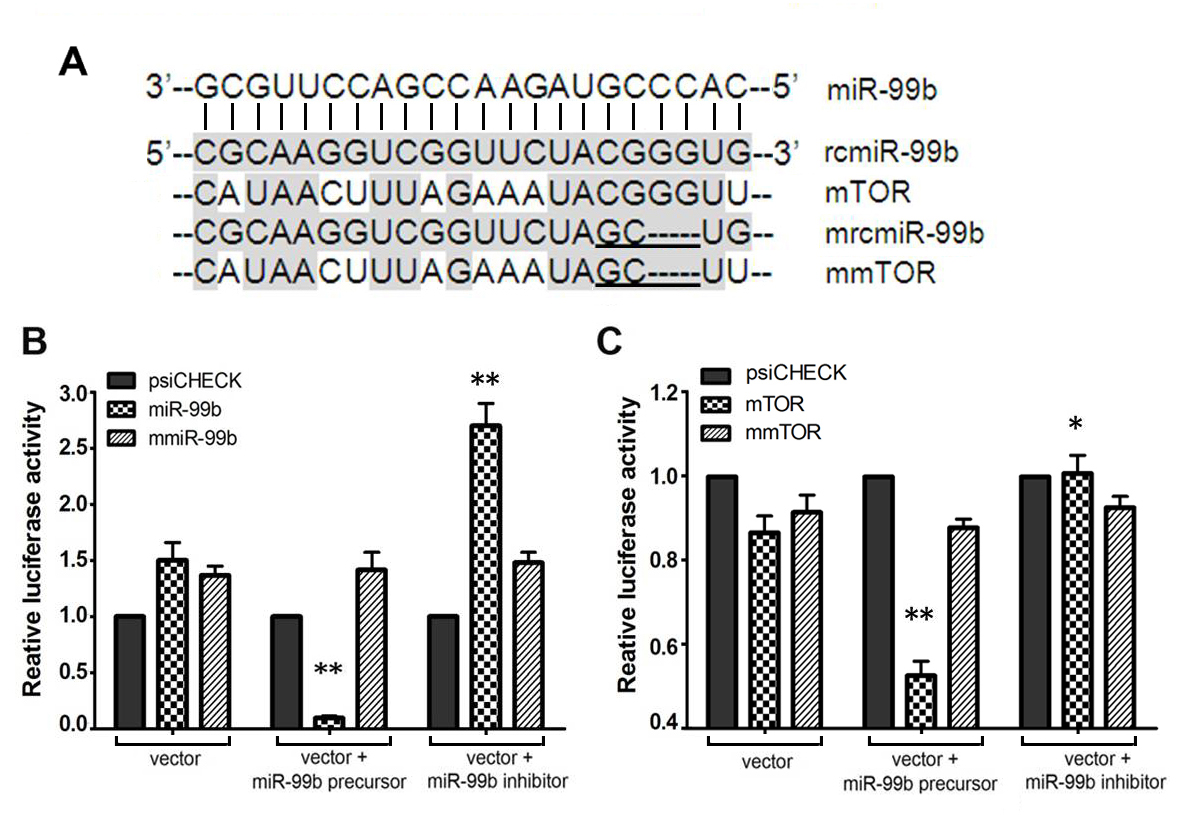
**

**Figure S1. mTOR is a target gene of miR-99b.** **(A)** Sequence alignment of miR-99b with reverse complementary miR-99b (rcmiR-99b, as the positive control), mTOR, mutant rcmiR-99b (mrcmiR-99b) and mutant mTOR (mmTOR); mutant nucleotides are underlined. **(B-C)** Dual-luciferase reporter vectors were constructed with rcmiR-99b/mrcmiR-99b and mTOR-3’-UTR/mmTOR-3’-UTR cloned between *Not* I and *Xho* I sites in psiCHECK plasmid and murine macrophage RAW264.7 cells were transfected with the vectors alone or in the presence of miR-99b precursor or inhibitor. Vectors containing rcmiR-99b and mrcmiR-99b were used as controls. *Renilla* luciferase (RLuc) activity was measured and normalized to *Firefly* luciferase (FLuc), and recombinant vectors were normalized to empty vector. *P < 0.05 and **P < 0.01 vs. plasmid alone group. Data are representative of three experiments.
